# Adequacy of Anaesthesia for Nociception Detection during Vitreoretinal Surgery

**DOI:** 10.3390/life13020505

**Published:** 2023-02-11

**Authors:** Michał Jan Stasiowski, Aleksandra Pluta, Anita Lyssek-Boroń, Ewa Niewiadomska, Lech Krawczyk, Dariusz Dobrowolski, Beniamin Oskar Grabarek, Magdalena Kawka, Robert Rejdak, Izabela Szumera, Anna Missir, Przemysław Hołyś, Przemysław Jałowiecki

**Affiliations:** 1 Chair and Department of Emergency Medicine, Faculty of Medical Sciences in Zabrze, Medical University of Silesia, 41-200 Sosnowiec, Poland; 2 Department of Anaesthesiology and Intensive Therapy, 5th Regional Hospital, 41-200 Sosnowiec, Poland; 3 Department of Ophthalmology with Paediatric Unit, 5th Regional Hospital, 41-200 Sosnowiec, Poland; 4 Department of Ophthalmology, Faculty of Medicine in Zabrze, Academy of Silesia, 41-800 Zabrze, Poland; 5 Department of Epidemiology and Biostatistics, School of Public Health in Bytom, Medical University of Silesia in Katowice, 41-902 Bytom, Poland; 6 Chair and Clinical Department of Ophthalmology, Faculty of Medical Sciences in Zabrze, Medical University of Silesia in Katowice, 40-760 Katowice, Poland; 7 Department of Histology, Cytophysiology and Embryology, Faculty of Medicine in Zabrze, Academy of Silesia, 41-800 Zabrze, Poland; 8 Department of General Ophthalmology, Medical University of Lublin, 20-059 Lublin, Poland

**Keywords:** vitreoretinal surgery (VRS), nociception detection, general anaesthesia (GA), adequacy of anaesthesia (AoA), intraoperative rescue opioid analgesia (IROA), surgical pleth index (SPI)

## Abstract

Vitreoretinal surgery (VRS) is one of the most widely performed precise procedures in ophthalmic surgery; the majority of cases are carried out under regional anaesthesia (RA) only. However, in specific situations (such as when the patient fails to cooperate with the operator for various reasons), general anaesthesia (GA), alone or in combination with GA (combined general–regional anaesthesia, CGR), is the only safe way to perform VRS. While monitoring the efficacy of an intraoperative rescue opioid analgesia (IROA) during surgery (assessing the adequacy of anaesthesia (AoA)) may be challenging, the surgical pleth index (SPI) is a useful tool for detecting the reaction to noxious stimuli and allows for the rational titration of opioid analgesics (AO) during surgery. The current study investigated the influence of the SPI-based titration of fentanyl (FNT) in combination with various pre-emptive analgesia (PA) techniques on intraoperative pain perception during various stages of VRS performed under AoA. A total of 176 patients undergoing VRS under GA were enrolled in the study. They were randomly assigned to one of the five following study arms: Group GA (control group)—patients who received general anaesthesia alone; Group PBB—GA with preprocedural peribulbar block (with 0.5% bupivacaine and 2% lidocaine); Group T—GA with preventive, topical 2% proparacaine; Group M—GA with a preprocedural intravenous infusion of 1.0 g of metamizole; and Group P—GA with a preprocedural intravenous infusion of 1.0 g of paracetamol. The whole procedure was divided in four stages: Stage 1 and 2—preoperative assessment, PA administration, and the induction of GA; Stage 3—intraoperative observation; Stage 4—postoperative observation. the SPI values were monitored during all stages. The occurrence of nociception (expressed as ∆SPI >15) during various manipulations in the surgical field was observed, as were cumulative doses of rescue analgesia, depending on the PA administered. During the course of VRS, rescue FNT doses varied depending on the stage of surgery and the group investigated. The majority of patients, regardless of their group allocation, needed complementary analgesia during trocar insertion, with Group GA patients requiring the highest doses. Likewise, the highest cumulative doses of IROA were noted during endophotocoagulation in Group GA. Preventive PBB and topical anaesthesia were proven to be most efficient in blunting the response to speculum installation, while topical anaesthesia and paracetamol infusion were shown to be more efficient analgesics during endophotocoagulation than other types used PA. In the performed study, none of the PA techniques used were superior to GA with FNT dosing under the SPI with respect to providing efficient analgesia throughout the whole surgery; there was a necessity to administer a rescue OA dose in both the control and investigated groups.

## 1. Introduction

Vitrectomy (vitreoretinal surgery (VRS)), or pars plana vitrectomy (PPV), is one of the most widely performed precise procedures in ophthalmic surgery. The development of ophthalmological techniques, which have resulted in a shortened length of time of the procedure, has supposedly made it possible to carry out the procedure under monitored anaesthesia care using a minimal alveolar concentration of sevoflurane (MAC), with regional anaesthesia (RA) being the most widely performed technique of pre-emptive analgesia (PA). There is an increasing number of patients requiring VRS, especially the elderly and those laden with various comorbidities (such as diabetes or arterial hypertension). However, the performance of VRS under PA with MAC may be impossible in selected elderly patients who fail to cooperate with medical staff during the operation due to the use of drugs that impair blood coagulation or the risk of eye globe perforation associated with a lack of patient cooperation due to a prolonged procedure. The necessity of implementing general anaesthesia (GA) entails the need to identify other PA techniques that can reduce the occurrence of major adverse events such as postoperative nausea and vomiting (PONV), hemodynamic instability due to inadequate pain control, and intolerable postoperative pain perception. In order to spare the use of intraoperative rescue opioid analgesics (IROAs), the concept of PA comes in handy [1,2]. The concept of PA is based on reducing the requirements for an IROA by blocking afferent noxious impulsation with RA or intravenous analgesia (for example, using acetaminophen or metamizole) to prevent central sensitization while maintaining immobilization with GA before surgical incision [3,4]. Monitoring the efficacy of an IROA during surgery constitutes a challenge during GA. While there have been many tools to monitor the depth of anaesthesia and hypnosis, such as response (RE), state entropy (SE), and neuromuscular transmission (NMT), there have not been any recent references to ideal techniques for the intraoperative monitoring of nociception, especially during GA. In everyday practice, IROA administration is usually based on the clinical judgement of the anaesthesiologist observing the fluctuation of haemodynamic parameters (heart rate and blood pressure) during VRS. The surgical pleth index (SPI) has gained popularity as a tool for monitoring the intraoperative nociception–anti-nociception balance during GA, RA, conscious sedation, or even the postoperative period [5,6]. The adequacy of anaesthesia (AoA), comprising the monitoring of RE, SE, NMT, and SPI, is crucial in assessing the quality of anaesthesia [7]. So far, we have published the current study findings regarding the influence of the employment of AoA guidance on GA with different PA techniques in patients undergoing VRS on the incidence of postoperative pain and perioperative haemodynamic stability [8,9,10,11] as preliminary reports. Currently, we investigate the intraoperative efficacy of various PA techniques, which are detected indirectly by observations of variations in SPI values, as an indication for IROA using FNT during various stages of VRS performed under AoA guidance. Pain is a very complex phenomenon. Thanks to the work of Melzack and Wall from 1965, who introduced the gate control theory of pain, it is widely known that a noxious signal can be modified at any stage of its conduction (upregulation or downregulation), which can result in the enhancement of impairment [12]. This ground-breaking study was a milestone in the perception of pain mechanisms and led to the development of pre-emptive analgesia (PA) [3,13].

Volatile anaesthetics may blunt or completely block the sympathetic response and, as a result, may diminish the haemodynamic response to noxious stimuli [14]. Therefore, during surgical manipulation in the operation field, patients receiving volatile anaesthetics may experience afferent noxious stimulation; however, such an adverse situation may not be illustrated in the fluctuation of haemodynamic parameters. At the time, the assessment of the quality of analgesia during GA is usually based solely on the observation of changes in the heart rate and blood pressure. This creates a contradictory situation when IROAs are not administered despite their necessity, consequently leading to inadequate intraoperative control with all its burdens, such as haemodynamic instability and acute postoperative pain. Therefore, assessing the adequacy of anaesthesia (AoA), including the quality of analgesia using SPI, to properly administer IROA cannot be overestimated. SPI values change in response to afferent noxious stimulations. This determines the monitoring of SPI to be a better tool for assessing the nociception–anti-nociception balance than relying on the fluctuation of haemodynamic parameters to determine the necessity of administering a dose of IROA [15,16,17]. The observation of the normalisation of the SPI value following IROA administration was proven to prevent the overdosage of the IROA [18,19].

In this way, the current main study report aims to indirectly identify whether there is a possibility of resignation from the performance of GA according to predictable manoeuvres in the operating field and the performance of the VRS under PA using MAC only as a result of efficient analgesia produced by the administration of PA of different types.

## 2. Materials and Methods

### 2.1. Ethics

Patients who met the inclusion criteria and were scheduled for elective primary PPV in the Department of Ophthalmology of Regional Hospital no. 5 in Sosnowiec, Poland, were asked to participate in the study. Patients with an American Society of Anaesthesiologists (ASA) score of I–III were enrolled after obtaining their written informed consent. Ethical approval for this study (KNW-1-183/N/9/K) was provided by the Ethical Committee of Medical University of Silesia on 29 September 2015. The project was registered in the Clinical Trial Registry (SilesianMUKOAiIT2, NCT02973581).

### 2.2. Subjects

Patients were randomly allocated into five groups: (1) Group GA—patients who received general anaesthesia alone; (2) Group T—patients who received preventive topical analgesia (3) Group PBB—patients who received PBB [14]; (4) Group M—patients who received PA using a single dose of 1 g of metamizole; and (5) Group P—patients who received PA using a single dose of 1 g of acetaminophen [8,10].

**Figure 1:**
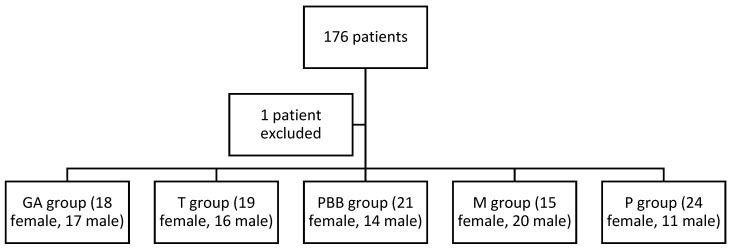
Allocation of subjects to five arms. GA group—patients who received general anaesthesia alone; T group—patients who received topical analgesia; PBB group—patients who received preprocedural peribulbar block; M group—patients who received metamizole; and P group—patients who received paracetamol.

Out of the 176 patients initially allocated to receive AoA-guided GA, 175 patients were analysed—one patient was excluded due to intraoperative technical problems with SPI monitoring (a result of unexpected arrhythmia). Figure 1 shows the division of the study group into the five subgroups mentioned above. A detailed description of the different groups, including procedures, can be found in our previous work, which dealt with the same group of patients [8,10].

The exclusion criteria were: withdrawing previous consent; blindness in the operated eye; a history of allergic reaction to local anaesthetics, metamizole, and acetaminophen; use of medications that alter blood coagulation; haematological diseases impairing blood clotting; pregnancy; drug or alcohol abuse; a history of neurological disease or a neurosurgical operation that would impair entropy EEG monitoring; and cardiac arrhythmia in ECG that might impair SPI monitoring.

### 2.3. Procedures

Throughout anaesthesia induction and surgery, standard monitoring procedures were utilised, and close attention was paid to vital parameters such as the non-invasive arterial pressure (NIBP), heart rate (HR), standard electrocardiography (ECG) lead II, arterial blood saturation (SaO2), fraction of inspired oxygen in the gas mixture (FiO2), fraction of inspired sevoflurane (FiAA), fraction of expired sevoflurane (FeAA), exhaled carbon dioxide concentration (etCO2), and MAC. The depth of anaesthesia was monitored with the entropy EEG (state and response entropy), intraoperative analgesia was guided with the surgical pleth index (SPI), and muscle relaxation was maintained using NMT monitoring (Carescape B650, GE, Helsinki, Finland) [8,10].

The whole procedure was divided into the following four stages:

STAGE 1: Upon admission to the operating theatre, the entropy EEG sensor (RE, SE) on the patient’s forehead, the pulse oximeter (SPI) on the contralateral finger to venous access, the NIBP cuff on the right arm, and the standard ECG on the patient’s back were placed according to the manufacturers’ suggestions. The first values were recorded. Depending on the group allocation, either a peribulbar block (PBB) was performed (group PBB), topical anaesthesia was administered (group T), or an acetaminophen/metamizole infusion was carried out (groups P and M, respectively). The PBB was performed by the same ophthalmologist, who had over 6 years of experience with the procedure, including at least 400 PBBs per year.

STAGE 2: To calculate the mean SPI value during Stage 2, SPI values were taken into account starting from 5 min after the placement of the laryngeal mask to the beginning of the sterilization of the orbita. This allowed for the calibration of the SPI sensor.

STAGE 3 (Intraoperatively): The SPI score was monitored online and recorded with a sampling frequency of 1 min. When the SPI value reached ∆SPI >15 points above the mean SPI value of Stage 2, a rescue dose of 1μg/kg of body weight of FNT was administered intravenously every 5 min until the SPI value decreased to value of the mean SPI of Stage 2. The duration of VRS was counted from the speculum installation to the speculum removal.

We assumed that the initial dose of FNT, 1 μg per kilogram of body weight, would produce sufficient analgesia before the installation of the speculum. In addition, in 2013, Gruenewald et al. [5] proposed a ∆SPI >10 or an absolute SPI value >50 as a predictor of inadequate analgesia. In other studies, only an absolute value of ∆SPI >50 was an indication for rescue analgesia [11]. In the methodology of the current study, a compromising protocol of ∆SPI >15, compared to the calculated baseline during Stage 2 and lasting at least one minute, was adopted as an indicator for rescue analgesia to avoid a potentially hazardous overdosage of FNT as a result of a potential miscalculation of the SPI score due to its variations. The PPV procedures were performed by the same ophthalmic surgeon, who had over 10 years of experience in VRS, performing over 400 vitreoretinal procedures a year.

STAGE 4 (Postoperatively): In the recovery room, all patients were monitored further (SPI, HR, SAP, MAP, DAP, and Sa02) by the anaesthesiology team, who were blinded to the patients’ group allocation. In line with postoperative hemodynamic parameters, the presence of adverse effects such as nausea, vomiting (PONV), level of sedation, and allergic reactions was monitored for each patient at the same time as pain assessment for 24 h. In the case of PONV, ondansetron (Ondansetron Accord, Accord Healthcare Limited, Devon, UK) was administered intravenously in a single dose of 4 mg. The Optylite solution, at a dose of 5 mL/kg of body weight, was infused in the case of MAP <65 mmHg. The patients received oxygen at a rate of 3 L/min via nasal cannulae. The patients were asked to rate their perception of pain intensity using the numeric pain rating scale (NRS), which ranges from 0 (no pain) to 10 (maximum pain), every 10 min. In the case of a pain perception of NRS >3, a standard dose of a non-steroidal, anti-inflammatory drug was administered according to contemporary guidelines on acute pain treatment issued by the Polish Society of Anaesthesiologists [3,12]. SPI values were monitored online, and the mean SPI values were recorded with a sampling time of 1 min (trends in the software provided by the producer). NRS and SPI values were recorded for acute pain (NRS 7–10), average pain (NRS 4–6), and mild pain (NRS 0–3) perception intervals. The patients were observed and monitored in the recovery room for at least 30 min until transfer from the recovery room to the Department of Ophthalmology. The monitoring and data recording were then ceased.

### 2.4. Statistical Analysis

Statistical analysis was carried out using the licensed version of the STATISTICA 12PL statistical software (Statsoft, Cracow, Poland), using a statistical significance threshold of (*p*) < 0.05. In the first step, we assessed whether the distribution of the numerical data conformed to the assumptions of a normal distribution by performing the Shapiro–Wilk test. If the assumptions of the Shapiro–Wilk test were met (*p* > 0.05), analyses were conducted using parametric tests: a one-way analysis of variance (ANOVA) preceded by Lene’s test of homogeneity of variances and Tukey’s post-hoc test with Bonferroni correction. The result was then presented as the mean ± standard deviation. When the assumptions of the Shapiro–Wilk test were not met (*p* < 0.05), non-parametric tests, i.e., the Kruskal–Wallis rank variance analysis and Dunn’s post-hoc test, were used for further statistical analyses. We then presented the data as the median. Regarding nominal data, their distribution was presented as the number of cases in the series and the percentage (percentage) of the total. The Chi-square test (χ^2^) was used in the statistical analysis of nominal data.

## 3. Results

The investigated group consisted of 97 (55.4%) women and 78 (44.6%) men. The anthropometric data of patients who underwent the final analysis are shown in Table 1. There were no statistically significant differences among groups in terms of age, height, weight, or BMI.

**Figure 2:**
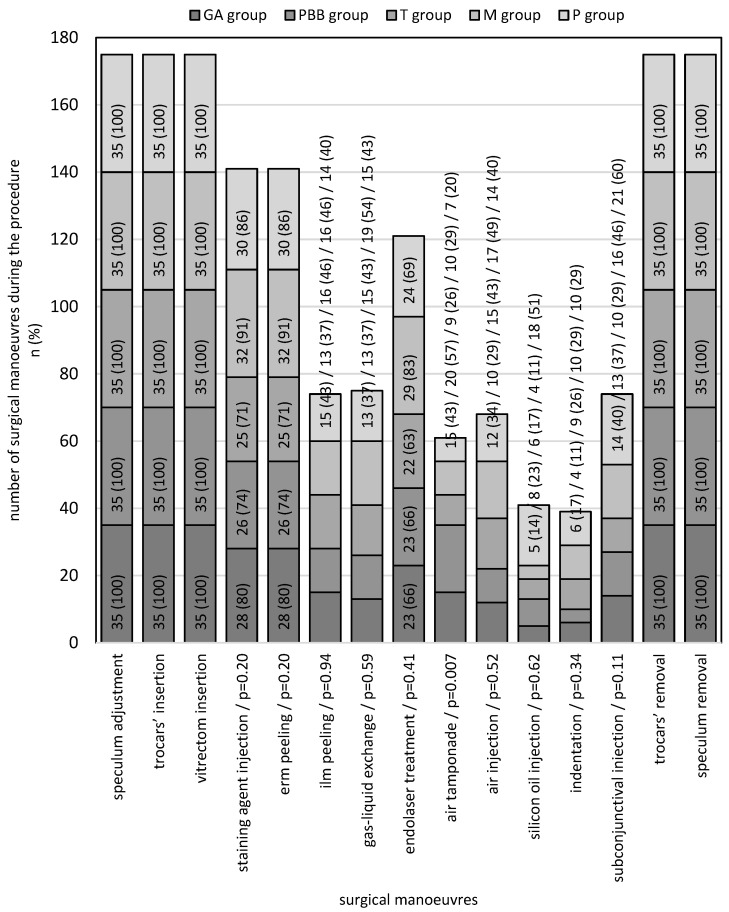
The frequency of surgical manoeuvres (numbers and percentages) during the procedure; *p*-values by the χ^2^ test of independence.

To ensure the homogeneity of the groups in further steps, we analysed the frequency of specific indications for surgery (Table 2) and the frequency of surgical manoeuvres during the procedure (Figure 2) among the groups. There was only one statistically significant difference, which concerned pseudophakia—such an indication to perform PPV was noted six times in the PPB group, while there was no patient in the P group with such a diagnosis. Moreover, aphakia was indicated for PPV in three patients in the P group and one in the PBB group, while no other patients were noted to have such a diagnosis in the remaining groups. However, these differences did not reach a level of statistical significance (*p* = 0.05).

The χ^2^ test of independence did not show any statistically significant difference except for the use of an air tamponade—this manoeuvre was more frequent in the PBB group than the other groups.

**Figure 3:**
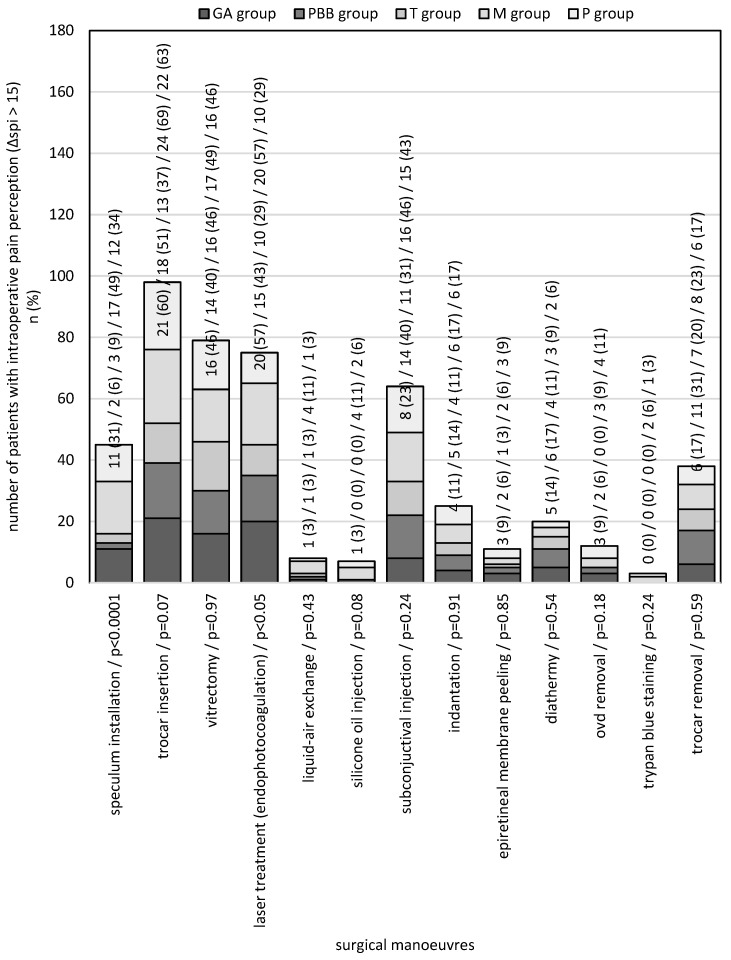
Number of patients with intraoperative pain perception (∆SPI >15) during certain manipulations in the surgical field; *p*-values by the χ^2^ test of independence.

As the current study focuses on intraoperative pain perception, we observed the occurrence of nociception (expressed as ∆SPI >15) during various manipulations in the surgical field (Figure 3). More than a quarter of the total patients (25.7%) reacted to speculum installation; the performance of PBB was proven to be most efficient in blunting this response as an ∆SPI >15 during this manoeuvre was noted in only two patients in the PBB group. This was similar to the T group (three patients). On the contrary, metamizole appeared to be least effective in preventing pain upon speculum installation as in 17 cases in the M group there was an increase in SPI >15.

Laser treatment (endophotocoagulation) appeared to be another strong noxious stimulus that should be taken into consideration during VRS; nearly half of the total patients (42.9%) reacted with an elevated SPI. Surprisingly, topical anaesthesia (T group) and acetaminophen (P group) were shown to be more efficient analgesics than PBB (PBB group) and metamizole (M group): 28.6% vs. 42.9% vs. 57.1%, respectively. In the control group (GA group), the efficiency of applied analgesia was the same as in the M group.

In the means of effective analgesia during trocar insertion, patients allocated to the T group needed the highest dose of rescue FNT during trocar insertion and while removing vitreous humour (vitrectomy), as shown in Table 3, whereas during other manipulations, the doses of rescue FNT were similar in every investigated group. Although the ∆SPI was usually >15 during subconjunctival injection, no rescue FNT was administered because it was a short final stage and the SPI value immediately reversed to the former value.

During the course of the whole procedure, the rescue FNT doses varied depending on the stage of surgery and investigated group (Table 4). Trocar insertion seemed to be most noxious as the majority of patients, regardless of their group allocation, needed complementary analgesia: almost half of the patients from the GA group (42.9%) required a dose of 100–150 μg FNT, while only one patient from the P group required such a dose. Higher doses of rescue FNT were also noted during vitrectomy in the T group, especially in comparison to the PBB and P group. Laser endophotocoagulation required a mean dose of rescue FNT of 100–150 μg in nine patients from the GA group and, in comparison, in only three patients from the M and P groups. Surprisingly, pain stimulus caused by the indentation of the eye globe seemed be the biggest issue in the PBB group, as those patients required higher rescue doses of FNT than other groups.

## 4. Discussion

In the current study, we performed a detailed analysis of the necessity of IROA administration using FNT, even if GA was performed, during certain surgical manipulations during VRS. We aimed to identify whether the employment of certain anaesthetic regimens, precisely described as study arms, could result in such a quality of deep, intraoperative analgesia that IROA using FNT was not needed and so that, in the next step, resignation with GA could possibly be taken into consideration for performing VRS under PA, especially in the PBB group. In the main report of the current study, we aimed to analyse intraprocedural pain perception (expressed as ∆SPI >15) during certain manipulations in the surgical field to determine whether the specific PA techniques may possibly produce sufficient analgesia during certain manipulations in the operation field such that AoA monitoring for GA could be abandoned and replaced by PA with MAC, even entailing IROA with FNT in doses <2 mcg/kg that are, in the majority of cases, perceived safe for awake patients. Only a few studies were found that provided such detailed analyses, but these were only based on patients’ overall satisfaction scores, indirectly indicating subjective phenomena of intraoperative perception of nociception at certain stages of surgical manipulations.

Despite the group allocation, IROA with FNT was administered in 45 patients (25.7%). These patients were mainly from the GA, M, and P groups, and received a dose lower than 2 mcg/kg to suppress the afferent nociceptive stimulation as a result of insufficient PA. Therefore, assumingly, the performance of PA with MAC instead of AoA-guided GA could be a safe alternative during this surgical manipulation in the operation field. Numerous studies reported the efficacy of TA or PBB during speculum installation [20,21,22,23,24], with a preference for PBB over TA [25]; this is similar to the findings of the current study.

Numerous studies proved trocar insertion to most often produce painful discomfort, regardless of anaesthesia performed prior to surgery in awake patients [25]. A similar observation was made in the current study, in which in 98 patients, in spite of their group allocation (56%), an increase in ∆SPI >15 was observed as an indication for rescue IROA with FNT as a result of insufficient PA. Although the results were statistically insignificant, the ∆SPI >15, reflecting insufficient analgesia, was observed in more than half of the patients from groups GA, PBB, M, and P. It is worth underlining that, in 18 cases (51.4%) in the PBB group, there was a necessity of IROA with FNT, indicating an ineffective PBB. The mean value of a cumulative dose of IROA with FNT was comparatively high in every investigated group, although the dose was not high enough to produce hypoventilation/apnoea if PA with MAC was performed instead of AoA-guided GA. However, in the T group, the incidence of ∆SPI >15 was the lowest and occurred in only 13 cases (37.1%). This stands in opposition to various studies available in the literature, which showed an inability of topical anaesthesia to prevent discomfort or pain upon trocar insertion [26].

In 79 cases (45.1%), despite the group allocation, a ∆SPI value >15 as an indicator of insufficient analgesia produced by PA during vitrectomy manipulations was noted, but only in y of these cases could the cumulative dose of IROA with FNT possibly result in hypoventilation/apnoea if the VRS was performed under PA with MAC (Table 4). Interestingly, in each patient with insufficient analgesia allocated to the PBB group, the dose of FNT was lower than 2 mcg/kg, which could produce adverse events in the case of abandonment of AoA-guided GA, leaving only PBB with MAC.

In 75 cases (42.9%), despite the group allocation, a ∆SPI value >15 (an indicator of insufficient analgesia produced by PA) were observed in the laser groups (GA and M vs. T and P) during the occurrence of nociceptive afferent stimulation. In 20 patients in the GA and M groups, this manipulation in the surgical field was recognised as nociceptive. This was demonstrated yet again by a peak in the ∆SPI value >15. On the contrary, in the T group and P group, there were only 10 such cases. The mean value of a cumulative dose of IROA with FNT was similar in both the control and studied groups (and was not statistically significant). The number of patients requiring a dose of IROA with FNT, which could possibly induce hypoventilation or apnoea in the case of possible performance of PA with MAC instead of AoA-guided GA being utilized, was eight cases, constituting a significant number by anaesthesiological standards (Table 4). Interestingly, during laser treatment (endophotocoagulation), insufficient analgesia produced by PBB was noted in 15 cases (33%), which also constitutes a significant number by anaesthesiological standards. However, only in one case could the dose of IROA possibly result in hypoventilation/apnoea if the treatment (endophotocoagulation) was noted. Statistically significant differences between grVRS were found under PBB with MAC. Contrary to current study findings, the current literature on PBB may provide sufficient analgesia for retinal panphotocoagulation. PA using oral paracetamol did not significantly reduce the pain associated with laser treatment, as in the current study [27].

Similar to speculum installation and trocar insertion, the demand for IROA based on observations of SPI value fluctuations was within a safe dosage during indentation despite the group allocation, although 25 patients (14.3%) required supplementary analgesia.

It is worth mentioning that, during the subconjunctival injection of PA, 64 patients (36.6%), despite their group allocation, did not produce sufficient analgesia and a ∆SPI >15 was observed. Most interestingly, in 14 patients, the utility of PBB was ineffective during this type of manipulation in the surgical field; however, at this time, IROA with FNT was not administered due to the short time duration of this manipulation. The same was true during trocar removal, which was perceived as noxious by 38 patients (21.7%).

It must be kept in mind that the administration of IROA, even if necessary, may inevitably result in serious adverse events [28]. We kept in mind that the administration of IROA with FNT during monitored anaesthesia care (MAC) in awake patients without GA was proven to be able to result in dangerous side effects such as chest wall rigidity [29] with resultant hypoventilation, known as wooden chest syndrome (WCS).

Therefore, intraoperatively detected insufficient analgesia, despite PA and GA requiring IROA with FNT in doses administered during MAC, could inevitably result in some of the above-mentioned adverse events, especially in the case of IROA with FNT >2 μg/kg body weight at the stage involving manipulations with vitrectomy or endophotocoagualtion. Moreover, according to the current study findings, during stages such as trocar insertion, vitrectomy manoeuvres, endophotocoagulation sessions, indentation, and/or subconjunctival injection, if the cumulative dose of FNT during the VRS lasted approximately an hour (as in the current study) and reached a dose of >2 μg/kg body weight in a patient, especially when ∆SPI values of >15 were observed during numerous stages in the individual patient and required the administration of IROA in a dose of 1 μg/kg body weight of FNT each time (according to the study protocol), the above-mentioned hazard may be unlikely.

Our study has some limitations. As in other studies concerning the utility of AoA guidance for GA in patients undergoing different surgical procedures, we adopted a protocol in which IROA was administered in the case of ∆SPI >15 [8,9,10,30,31,32] to avoid miscalculation and the possibly hazardous overdosage of IROA. This was already precisely described with a more strict protocol, although the current literature reports that ∆SPI >10 or any SPI >50 constitute an indication for IROA. Additionally, in patients allocated to the PBB group, the anaesthetic mixture contained bupivacaine and lidocaine to ensure the fast onset of PBB and a long-lasting perioperative analgesia as we did not expect globe akinesia due to the use of general anaesthesia; other local anaesthetic mixtures could possibly offer more promising results [33]. This requires further study in combination with the AoA-guided GA currently performed in our centre. Finally, insufficient analgesia may not always be reflected in tachycardia or elevated blood pressure because volatile anaesthetics (such as the sevoflurane used in the current study) may blunt or completely block the sympathetic response and consequently diminish the haemodynamic response to noxious stimuli. This can be reflected in a lack of variation in SPI values [6]. Therefore, the number of incidences of insufficient analgesia during different stages of performed VRS in the current study is certainly underreported. However, the proportions are hopefully equal between the techniques of PA employed in study groups.

In summary, although VRS has been deemed a fairly painless procedure associated with minimal tissue disruption, the current study findings indirectly prove that individual patients may still suffer from unacceptable intraoperative nociception perception during certain manoeuvres in the surgical field. These entailed the administration of IROA in each case of ∆SPI >15, according to the study protocol. In numerous patients in the current study, despite their group allocation, if the VRS was performed under PA with MAC instead of GA with AoA monitoring, the administration of IROA under the observation of ∆SPI values >15 could possibly induce opioid-related side effects (even with apnoea) or present potential intraoperative pain perception, diminishing the patients’ satisfaction with the medical services and the comfort of ophthalmic surgeons. Although PA was proven in multiple studies to be splendidly efficient in not only providing adequate postoperative analgesia but also in diminishing the need for IROA, in the performed study analysis, surprisingly, none of the PA techniques used proved to be superior in the AoA monitoring of GA, especially with the SPI guidance of IROA using FNT, in terms of providing efficient analgesia throughout the whole surgery. Keeping in mind the potential adverse events associated with every PA proposed (such as systemic local anaesthetic toxicity in case of RA, local allergic reactions during topical anaesthesia, agranulocytosis or anaphylaxis due to metamizole, or hepatotoxicity following acetaminophen administration), the economic frugality of their administration should be thoroughly considered against cost–risk analysis. Notwithstanding the abovementioned concerns, the monitoring of GA using the AoA, especially with respect to IROA administration under SPI guidance, seems to remain an irreplaceable method of assessment of AoA during surgery for the reasons outlined above.

## 5. Conclusions

In the current study, the introduction of monitoring GA using the AoA, especially to guide the administration of IROA using observations of variations of SPI values in patients undergoing VRS, revealed that no PA regimen is able to provide such a quality of analgesia that intraoperative pain perception is completely blunted and, as a result, the performance of GA could possibly be reasonably abandoned in all cases.

In the current study, intraoperative pain perception during VRS, reflected by an intraoperative increase in the ∆SPI value >15 regardless of the PA technique used, was observed in most cases during trocar insertion, vitrectomy manoeuvres, endophotocoagulation sessions, indentation, and subconjunctival injection. Therefore, further studies are required to establish an anaesthetic regimen with different PA techniques that could inevitably lead to the possible abandonment of GA performance, which was proven to be efficient by observations of stable SPI values at all stages of VRS with no necessity to administer IROA. Studies of this kind are currently being carried out in our centre.

## Figures and Tables

**Table 1:** Anthropometric data of the patients in the studied groups.

Anthropometric Data	Total n = 175 (100)	GA Group n = 35 (20)	PBB Group n = 35 (20)	T Group n = 35 (20)	M Group n = 35 (20)	P Group n = 35 (20)	*p*-Value
Age [years]	64.5 ± 11.7 66 (13)	65.1 ± 10.8 67 (9)	66.8 ± 1.1 69 (13)	62.7 ± 13.3 65 (14)	61.9 ± 11.9 63 (14)	66.1 ± 9.9 67 (8)	0.25 ^a^
Height [cm]	165.8 ± 8.7 165 (12)	166.9 ± 8.6 168 (14)	165.9 ± 8.3 164 (12)	164.7 ± 10.3 164 (18)	168 ± 7.4 170 (14)	163.4 ± 8.7 160 (12)	0.18 ^a^
Weight [kg]	77.6 ± 15.9 75.5 (17)	83.4 ± 19.8 82 (20)	78.8 ± 16 75 (11)	77.1 ± 13.7 80 (21)	74.7 ± 14.9 74 (19)	74.1 ± 13.3 74 (22)	0.19 ^a^
BMI [kg/m^2^]	28.3 ± 5.4 27.5 (6.4)	29.9 ± 6.6 28.4 (5.3)	28.6 ± 5.1 27.1 (4.4)	28.5 ± 4.9 28.4 (7.3)	26.4 ± 4.6 25.3 (5.4)	27.9 ± 5.3 27.6 (7.7)	0.06 ^a^
Male/ Female	78 (44.6)/ 97 (55.4)	17 (48.6)/ 14 (51.4)	14 (40.0)/ 21 (60.0)	16 (45.7)/ 19 (54.3)	20 (57.1)/ 15 (42.9)	11 (31.4) / 24 (68.6)	0.26 ^b^

Results are presented as means ± standard deviations and medians (interquartile ranges). ^a^ One-way analysis of variance (ANOVA)/the Kruskal–Wallis test by rank. Nominal data are presented as numbers (percentages). ^b^ χ^2^ test of independence. BMI—body mass index.

**Table 2:** The frequency of specific indications for surgery.

Indications for Surgery	Total n = 175 (100)	GA Group n = 35 (100)	PBB Group n = 35 (100)	T Group n = 35 (100)	M Group n = 35 (100)	P group n = 35 (100)	*p*-Value
Vitreal haemorrhage	36 (20.6)	9 (25.7)	5 (14.3)	6 (17.1)	10 (28.6)	6 (17.1)	0.53 ^a^
Retinal detachment	55 (31.4)	7 (20.0)	12 (34.3)	12 (34.3)	12 (34.3)	12 (34.3)	0.60 ^a^
Macular hole	25 (14.3)	6 (17.1)	4 (11.4)	6 (17.1)	3 (8.6)	6 (17.1)	0.77 ^a^
Epiretinal membrane	146 (83.4)	33 (94.3)	26 (74.3)	26 (74.3)	31 (88.6)	30 (85.7)	0.12 ^a^
Macular oedema	11 (6.3)	2 (5.7)	2 (5.7)	3 (8.6)	2 (5.7)	2 (5.7)	0.97 ^a^
Ocular trauma	3 (1.7)	0 (0)	1 (2.9)	1 (2.9)	1 (2.9)	0 (0)	0.80 ^a^
Aphakia	4 (2.3)	0 (0)	1 (2.9)	0 (0)	0 (0)	3 (8.6)	0.05 ^a^
Pseudophakia	11 (6.3)	1 (2.9)	6 (17.1)	3 (8.6)	1 (2.9)	0 (0)	0.03 ^a^
Multiple indications	99 (56.6)	25 (71.4)	18 (51.4)	20 (57.1)	13 (37.1)	23 (65.7)	0.46 ^a^

Nominal data are presented as numbers (percentages). ^a^ χ^2^ test of independence.

**Table 3:** Mean value of cumulative dose of rescue analgesia in patients with intraoperative pain perception during certain manipulations in the surgical field, when ∆SPI >15 was observed.

Cumulative Dose of Rescue FNT in Patients with Intraoperative Pain Perception (∆SPI >15)	Total n = 175 (100)	GA Group n = 35 (20)	PBB Group n = 35 (20)	T Group n = 35 (20)	M Group n = 35 (20)	P Group n = 35 (20)	*p*-Value
FNT—STAGE 3							
Speculum installation	25.6 ± 27.4 0 (50)	13.6 ± 23.4 0 (50)	25 ± 35.4 25 (50)	50 ± 0 50 (0)	38.2 ± 28.1 50 (50)	12.5 ± 22.6 0 (25)	<0.05 ^a^
Trocar insertion	52.6 ± 30.8 50 (0)	66.7 ± 32.9 50 (50)	44.4 ± 37.9 50 (50)	61.5 ± 30 50 (50)	47.9 ± 27.5 50 (0)	45.5 ± 21.3 50 (0)	0.07 ^a^
Vitrectomy	63.9 ± 48 50 (50)	71.9 + 57.6 50 (50)	53.6 + 41.4 50 (100)	90.6 + 41.7 100 (50)	64.7 + 46 50 (50)	37.5 + 38.7 50 (50)	<0.05 ^a^ T vs. P
Laser treatment (endophotocoagulation)	78.3 ± 50.4 50 (50)	85 ± 40.1 100 (50)	73.3 ± 45.8 50 (50)	85 ± 53 100 (50)	81.3 ± 68.3 50 (50)	60 ± 31.6 50 (50)	0.55 ^a^
Liquid–air exchange	-	-	-	-	-	-	-
Silicone oil injection	-	-	-	-	-	-	-
Subconjunctival injection	-	-	-	-	-	-	-
Indentation	39.6 ± 29.4 50 (50)	50 ± 0 50 (0)	70 ± 27.4 50 (50)	39.6 ± 29.4 50 (50)	33.3 ± 25.8 50 (50)	16.7 ± 25.8 0 (50)	0.21 ^a^
Epiretinal membrane peeling	-	-	-	-	-	-	-
Diathermy	-	-	-	-	-	-	-
OVD removal	-	-	-	-	-	-	-
Trypan blue staining	-	-	-	-	-	-	-
Trocar removal	-	-	-	-	-	-	-
Speculum removal	-	-	-	-	-	-	-

Results are presented as means ± standard deviations and medians (interquartile ranges). ^a^ One-way analysis of variance (ANOVA)/the Kruskal–Wallis test by rank.

**Table 4:** Detailed values of cumulative dose of rescue analgesia in patients with intraoperative pain perception during certain manipulations in the surgical field, when ∆SPI >15 was observed.

Cumulative Dose of Rescue FNT in Patients with Intraoperative Pain Perception (∆SPI >15)		Total n = 175 (100)	GA Group n = 35 (100)	PBB Group n = 35 (100)	T Group n = 35 (100)	M Group n = 35 (100)	P Group n = 35 (100)	*p*-Value
0	Speculum installation	23 (51.1)	8 (72.7)	1 (50)	0 (0)	5 (29.4)	9 (75)	0.09 ^a^
50–100		21 (46.7)	3 (27.3)	1 (50)	3 (100)	11 (64.7)	3 (25)	
100–150		1 (2.2)	0 (0)	0 (0)	0 (0)	1 (5.9)	0 (0)	
0	Trocar insertion	16 (16.3)	2 (9.5)	6 (33.3)	1 (7.7)	4 (16.7)	3 (13.6)	<0.05 ^a^
50–100		61 (62,2)	10 (47.6)	8 (44.4)	8 (61.5)	17 (70.8)	18 (81,8)	
100–150		21 (21.4)	9 (42.9)	4 (22.2)	4 (30.8)	3 (12.5)	1 (4.5)	
0	Vitrectomy	17 (21.5)	2 (12.5)	4 (28.6)	1 (6.3)	3 (17.6)	7 (43.8)	<0.05 ^a^
50–100		32 (40.5)	9 (56.3)	5 (35.7)	4 (25)	8 (47.1)	6 (37.5)	
100–150		23 (29.1)	3 (18.8)	5 (35.7)	8 (50)	4 (23.5)	3 (18.8)	
150–200		5 (6.3)	0 (0)	0 (0)	3 (18.8)	2 (11.8)	0 (0)	
>200		2 (2.5)	2 (12.5)	0 (0)	0 (0)	0 (0)	0 (0)	
0	Laser treatment (endophotocoagulation)	5 (6.8)	0 (0)	1 (6.7)	1 (10)	2 (10.5)	1 (10)	0.75 ^a^
50–100		36 (48.6)	9 (45)	8 (53.3)	3 (30)	10 (52.6)	6 (60)	
100–150		25 (33.8)	9 (45)	5 (33.3)	5 (50)	3 (15.8)	3 (30)	
150–200		2 (2.7)	1 (5)	0 (0)	0 (0)	1 (5.3)	0 (0)	
200–250		5 (6.8)	1 (5)	1 (6.7)	1 (10)	2 (10.5)	0 (0)	
>250		1 (1.4)	0 (0)	0 (0)	0 (0)	1 (5.3)	0 (0)	
0	Indentation	7 (29.2)	0 (0)	0 (0)	1 (33.3)	2 (33.3)	4 (66.7)	0.06 ^a^
50–100		15 (62.5)	4 (100)	3 (60)	2 (66.7)	4 (66.7)	2 (33.3)	
100–150		2 (8.3)	0 (0)	2 (40)	0 (0)	0 (0)	0 (0)	

Nominal data are presented as numbers (percentages). ^a^ χ^2^ test of independence.

## Data Availability

The data used to support the findings of this study are included within the article.
